# Transcriptome Analysis Reveals the Regulatory Networks of Cytokinin in Promoting Floral Feminization in *Castanea henryi*

**DOI:** 10.3390/ijms23126389

**Published:** 2022-06-07

**Authors:** Guo-Long Wu, Zhou-Jun Zhu, Qi Qiu, Xiao-Ming Fan, De-Yi Yuan

**Affiliations:** 1 Key Laboratory of Cultivation and Protection for Non-Wood Forest Trees, Ministry of Education, Central South University of Forestry and Technology, Changsha 410004, China; hopeoflong@163.com (G.-L.W.); csuftzzyy@163.com (Z.-J.Z.); qq264629@163.com (Q.Q.); 2 Key Lab of Non-Wood Forest Products of State Forestry Administration, Central South University of Forestry and Technology, Changsha 410004, China

**Keywords:** *Castanea*, CPPU, floral feminization, sex differentiation, endogenous phytohormone

## Abstract

*Castanea henryi* is a monoecious plant with a low female-to-male ratio, which limits its yield. The phytohormone cytokinin (CK) plays a crucial role in flower development, especially gynoecium development. Here, the feminizing effect of CK on the development of *C. henryi* was confirmed by the exogenous spraying of N-(2-chloro-4-pyridyl)-N’-phenylurea (CPPU). Spraying CPPU at 125 mg·L^−1^ thrice changed the male catkin into a pure female catkin, whereas at 5 mg·L^−1^ and 25 mg·L^−1^, only a part of the male catkin was transformed into a female catkin. A comparative transcriptome analysis of male catkins subjected to CPPU was performed to study the mechanism of the role of CKs in sex differentiation. Using Pearson’s correlation analysis between hormone content and hormone synthesis gene expression, four key genes, *LOG1*, *LOG3*, *LOG7* and *KO*, were identified in the CK and GA synthesis pathways. Moreover, a hub gene in the crosstalk between JA and the other hormone signaling pathways, *MYC2*, was identified, and 15 flowering-related genes were significantly differentially expressed after CPPU treatment. These results suggest that CK interacts with other phytohormones to determine the sex of *C. henryi*, and CK may directly target floral organ recognition genes to control flower sex.

## 1. Introduction

*Castanea henryi* (Skam) Rehd. et Wils. is an important woody food plant of the Fagaceae family. Its nuts are renowned for their sweet and glutinous taste and high nutritional value [1]. However, the low female-to-male flower ratio of *C. henryi*, with over two thousand male flowers arranged in the male catkins, only one bisexual catkin for every five male catkins and only two female flowers growing at the base of the bisexual catkins [2], has significantly limited its seed yield and industrial potential. Therefore, revealing the molecular mechanism of *C. henryi* sex determination is of great significance for improving its fruit yield.

Various stages of plant growth and development are controlled by a variety of phytohormones. Phytohormones, such as auxin, ethylene (ETH), cytokinin (CK) and gibberellin (GA), regulate sex differentiation. It is generally believed that CK, auxin and ETH are positive regulators of pistil development, whereas GA promotes the development of androecium organs [3,4,5,6]. Spraying CK on *Mercurialis annua* increases the number of female flowers [7]. Moreover, the exogenous spraying of 6-benzylaminopurine (6-BA) in *Jatropha curcas* can increase the total number of flowers on the catkin, induce bisexual flowers and improve the ratio of males to females [8]. Likewise, the fine-tuning of endogenous CKs can increase flower number and male-to-female ratios in *J. curcas* [9]. In C. henryi, CK is accumulated more in female flowers than in male flowers, and the exogenous application of CK increases the number of female flowers. However, the mechanisms through which CK regulates sex differentiation remain unknown.

In the present study, we proved that CK is a crucial regulator of sex determination in *C. henryi*. The feminization effect of N-(2-chloro-4-pyridyl)-N’-phenylurea (CPPU) treatment, which has been reported to be 10–100 times more effective than 6-BA [10] and has a considerable feminization effect on *Vitis amurensis* [11] and *Litchi chinensis* [12], was detected at different spray times (once, twice and thrice) and spray concentrations (5 mg·L^−1^, 25 mg·L^−1^, and 125 mg·L^−1^). Thus, the levels of nine endogenous hormones were determined, and a comparative transcriptomic analysis was performed of male catkins under different treatments in *C. henryi* to identify the regulation networks that participate in promoting the floral feminization in *C. henryi*. The present study provides a basic framework for the regulatory networks of sex determination in *C. henryi,* and transcriptome data provide a theoretical basis for *C. henryi* genetic diversity analysis and molecular-assisted breeding.

## 2. Results

### 2.1. Feminization Effects of CPPU Treatment on Floral Development in C. henryi

The catkins of *C. henryi* are divided into two types: male and bisexual. The bisexual catkins have upper and lower parts, with male flowers on top and 1–3 protruding female flowers at the bottom. Exogenous CPPU treatment strongly induced female flowers in male catkins at the location of the male flowers.

**Figure 1:**
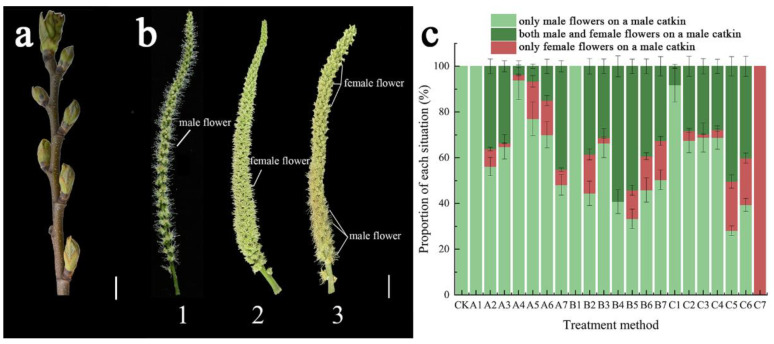
(**a**) The state of the first N-(2-chloro-4-pyridyl)-N’-phenylurea (CPPU)-treated catkins; 1. Only male flowers on male catkin; 2. Only female flowers on female catkin; 3. Both male and female flowers on male catkin; (**b**) The sex of flowers on male catkins in *Casatanea henryi*; (**c**) The proportions of different sexes of the flower on the male catkins under different treatments of *Casatanea henryi*. All scale bars = 10 mm.

**Figure 2:**
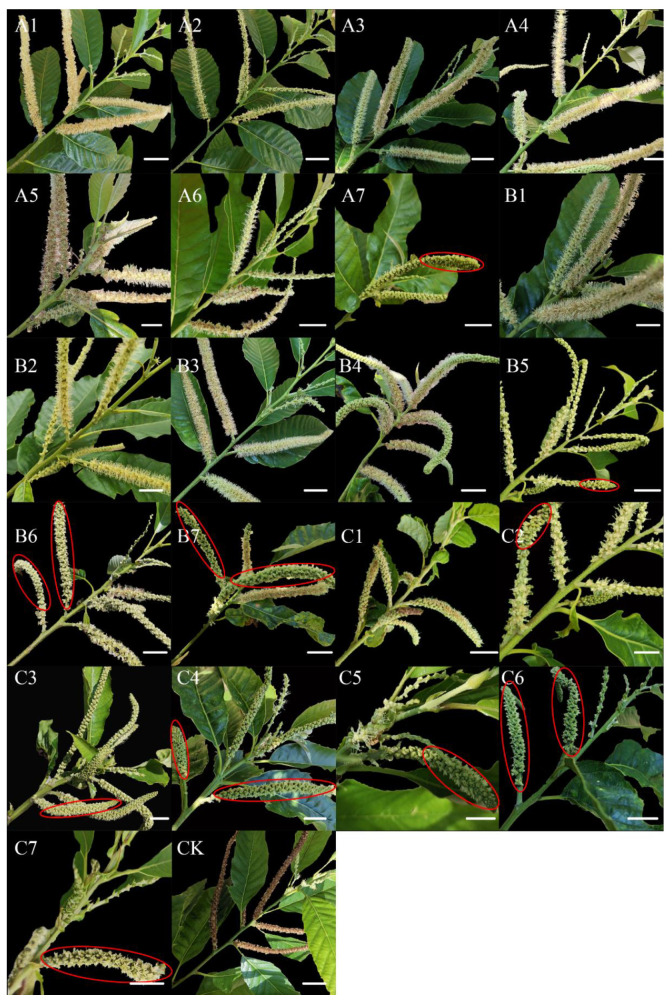
The male catkin of *Casatanea henryi* under different treatments. **A1**–**C7** represent different treatment methods. **CK** is the control group of pure water treatment. The female flowers are circled in red. All scale bars = 10 mm.

The sex of flowers on male catkins was investigated in three situations (Figure 1b): (1) only male flowers on a male catkin; (2) only female flowers on a male catkin; and (3) both male and female flowers on a male catkin; the proportion of each situation was counted (Figure 1c). The feminization effect increased with increasing concentrations of CPPU, and it also changed with the treatment time and frequency (Figure 2). The treatment group C7 exhibited the strongest feminization effect, with the transformation of all the male flowers on the male catkin into female flowers (Figure 2).

### 2.2. Effect of CPPU Treatment on the Content of Endogenous Hormones in C. henryi Male Catkins

**Figure 3:**
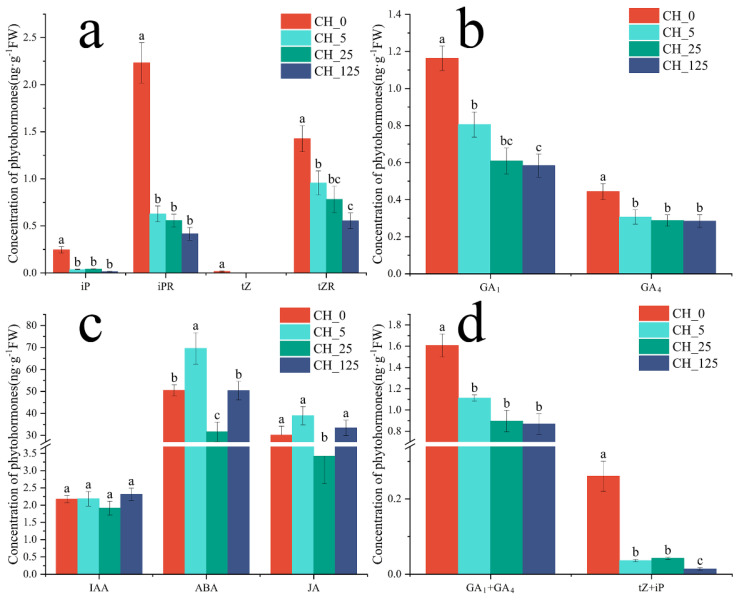
Effects of different concentrations of N-(2-chloro-4-pyridyl)-N’-phenylurea (CPPU) on endogenous hormones of *Castanea henryi* male catkins. In the figure, CH_0, CH_5, CH_25 and CH_125 represent different CPPU treatment concentrations (mg·L^−1^). (**a**) The levels of endogenous isopentenyladenine (iP), isopentenyladenosine (iPR), zeatin (tZ) and trans-zeatin riboside (tZR) in the male catkins of *C. henryi* treated with different concentrations of CPPU. (**b**) The levels of endogenous GA_1_ and GA_4_ in the male catkins of *C. henryi* treated with different concentrations of CPPU. (**c**) The levels of endogenous Auxin (IAA), Abscisic acid (ABA), and jasmonate (JA) in the male catkins of *C. henryi* treated with different concentrations of CPPU. (**d**) The levels of endogenous tZ + iP and GA_1_ + GA_4_ in the male catkins of *C. henryi* treated with different concentrations of CPPU. The values represent the means ± standard deviations (n = 3). Student’s *t*-test was used for the statistical analyses. Small letters mean a significant difference at the 0.05 level.

The levels of the four measured cytokinins, isopentenyladenine (iP), isopentenyladenosine (iPR), zeatin (tZ) and trans-zeatin riboside (tZR), were significantly lower in the treatment group than in the control group (Figure 3a). The levels of the two gibberellins observed, GA_1_ and GA_4_, were significantly lower in the treated group than in the control group (Figure 3b). Auxin (IAA) levels did not differ significantly between the control and treatment groups. Abscisic acid (ABA) levels showed no clear trend among the treatment groups. The level of jasmonate (JA) in the CH_25 group was significantly lower than it was in the other groups (Figure 3c).

In plants, tZ and iP are the primary bioactive CKs [13], and GA_1_ and GA_4_ are the primary bioactive gibberellins [14]. Hence, the levels of tZ + iP and GA_1_ + GA_4_ were determined (Figure 3d). The results show that, after CPPU treatment, the levels of tZ + iP and GA_1_ + GA_4_ were significantly lower in the treatment groups than they were in the control groups.

### 2.3. Transcriptome Sequencing of the Male Catkins of C. henryi in Response to CPPU Treatment

The induction of abundant female flowers on the male catkin by the CPPU treatment suggests that CK is a key factor in regulating sex differentiation in *C. henryi*. Subsequently, a comparative transcriptome analysis of male catkins treated with CPPU was performed to investigate the mechanism of action of CKs in the sex differentiation of *C. henryi*.

**Figure 4:**
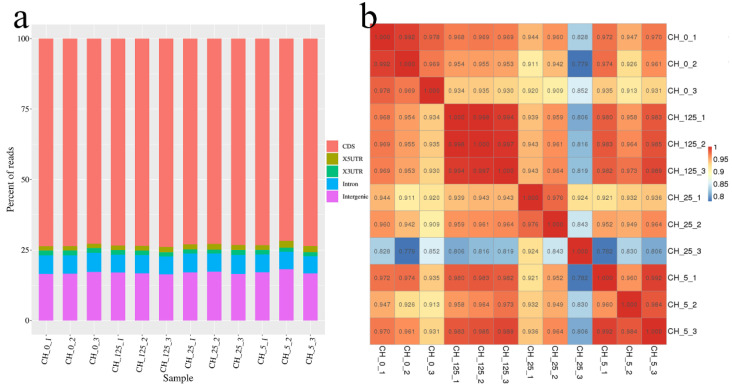
(**a**) Distribution of all samples of gene functional elements. (**b**) Pearson correlation coefficients between samples.

In *C. henryi*, flower buds are located in the catkin. Two weeks after the third spray, male catkins from different treatments were sampled. The samples from CK, A7, B7 and C7 treatments in Table 5 were named CH_0, CH_5, CH_25 and CH_125, respectively. To identify early response genes involved in CK-regulated sex determination, three replicates of four groups of samples (three different concentrations of CPPU-treated and pure water-treated male catkins) were collected for subsequent transcriptome sequencing and quantitative real-time polymerase chain reaction (RT-qPCR). Twelve cDNA libraries were constructed and sequenced using an Illumina high-throughput sequencing platform. After sequencing, the raw data were obtained, the unqualified sequences were filtered out to obtain clean data and statistics were performed to obtain basic information about the data (Table 1). The distributions of gene functional elements in all samples and the Pearson correlation coefficient analysis between each sample are shown in Figure 4.

### 2.4. Functional Annotation and Classification of Differentially Expressed Genes (DEGs)

**Figure 5:**
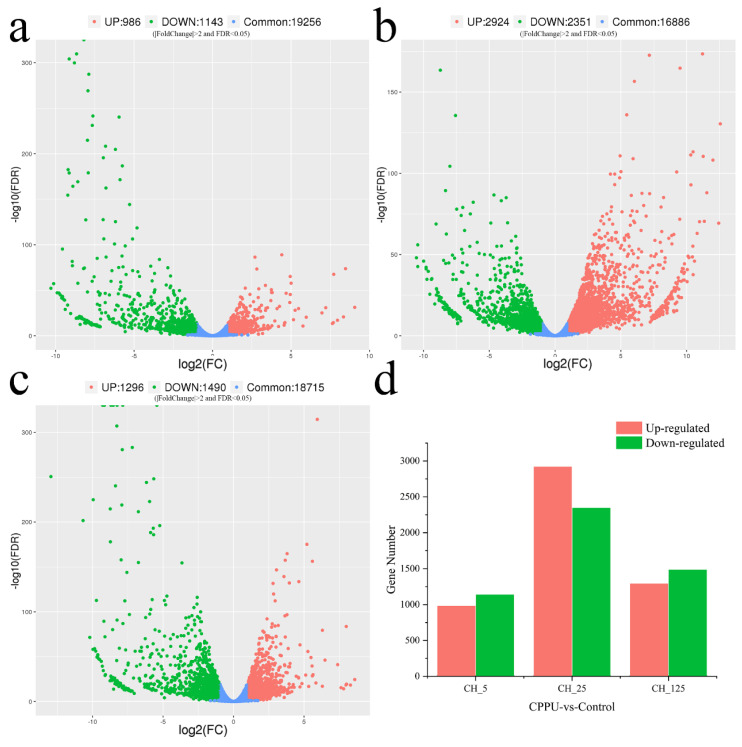
Differentially expressed genes (DEGs) of male catkin treated with three different concentrations of N-(2-chloro-4-pyridyl)-N’-phenylurea (CPPU) (5 mg·L^−1^, 25 mg·L^−1^, and 125 mg·L^−1^). (**a**–**c**) Differential gene expression maps drawn using the fragments per kilobase of exon per million reads mapped (FPKM) method. The red and green dots represent DEGs with |Fold change| > 2 and FDR < 0.05. (**d**) The number of DEGs. The red and green columns indicate up- and down-regulated DEGs, respectively.

**Figure 6:**
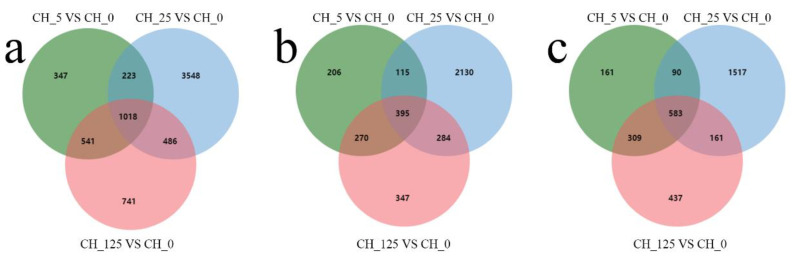
Venn diagrams of differentially expressed genes (DEGs) in different samples: (**a**) Venn diagram of all DEGs in different samples; (**b**) Venn diagram of up-regulated DEGs in different samples; (**c**) Venn diagram of down-regulated DEGs in different samples.

**Figure 7:**
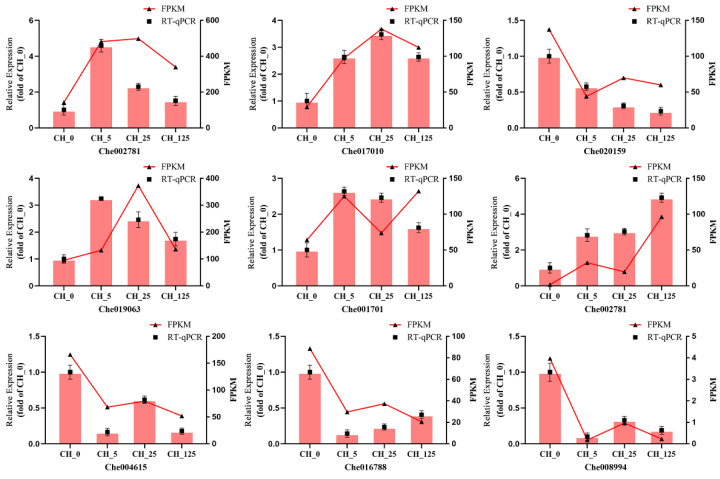
Validation of the expression patterns of differentially expressed genes selected from the RNA-seq analysis by RT-qPCR. The relative gene expression levels determined by RT-qPCR are shown on the left Y-axis, whereas the selected gene expression levels derived by the fragments per kilobase per million reads method are shown on the right Y-axis. The reference gene is GAPDH, and the values represent the means ± standard deviations (n = 3).

To compare the differences in gene expression between different samples, the expression levels of significantly different genes in all samples were extracted into a list, which was then used to draw a volcano plot of differentially expressed genes. The differences of gene expression levels were plotted with colored dots, and there were separately 2129, 5275 and 2786 genes differentially expressed after three different concentrations (5 mg·L^−1^, 25 mg·L^−1^ and 125 mg·L^−1^) of CPPU treatment (Figure 5). To visualize the similarity and overlap in the number of DEGs in different samples, Venn diagrams are used to show the number of shared and unique genes in different samples (Figure 6). To verify the accuracy of the transcriptome sequencing results, RT-qPCR was used to detect the expression levels of nine DEGs. The primer sequences for the reference gene (GAPDH) and the nine selected genes are listed in Table 2. The results show that the expression profiles of these genes are consistent with RNA-seq values (Figure 7).

**Figure 8:**
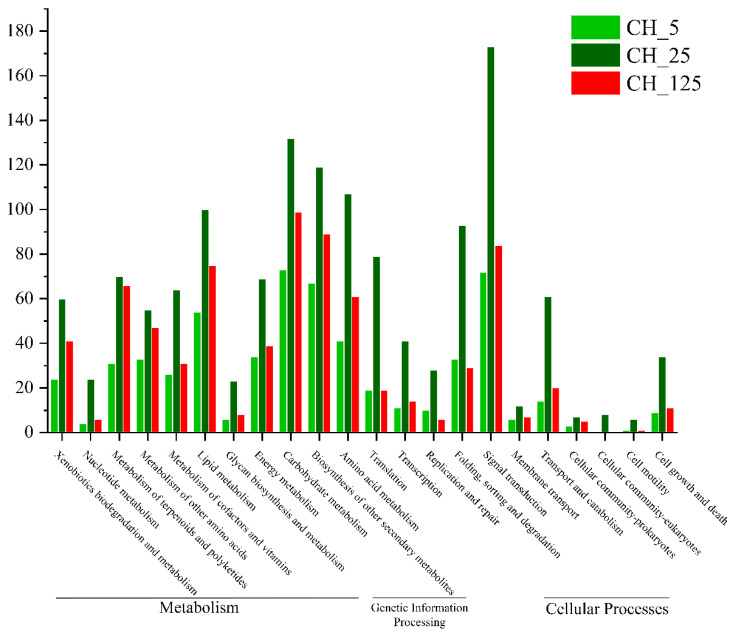
Kyoto Encyclopedia of Genes and Genomes (KEGG) analysis of differentially expressed genes in three different concentrations of N-(2-chloro-4-pyridyl)-N’-phenylurea (CPPU) treatment (5 mg·L^−1^, 25 mg·L^−1^, and 125 mg·L^−1^).

For an overall description of their biological functions, the DEGs were analyzed using the Kyoto Encyclopedia of Genes and Genomes (KEGG) and Gene Ontology (GO) analyses. The KEGG analysis divided the DEGs into 22 categories, among which categories of “Metabolism” (66%) and “Genetic information processing” (14%) were the most prominent (Figure 8). In addition, GO analysis divided the DEGs into “cellular process”, “metabolic process”, “cell”, “cell part” and “catalytic activity”, which were related to the basic biological function of CK to promote cell growth and metabolism.

### 2.5. DEGs Related to Endogenous CK Biosynthesis and Signaling

**Figure 9:**
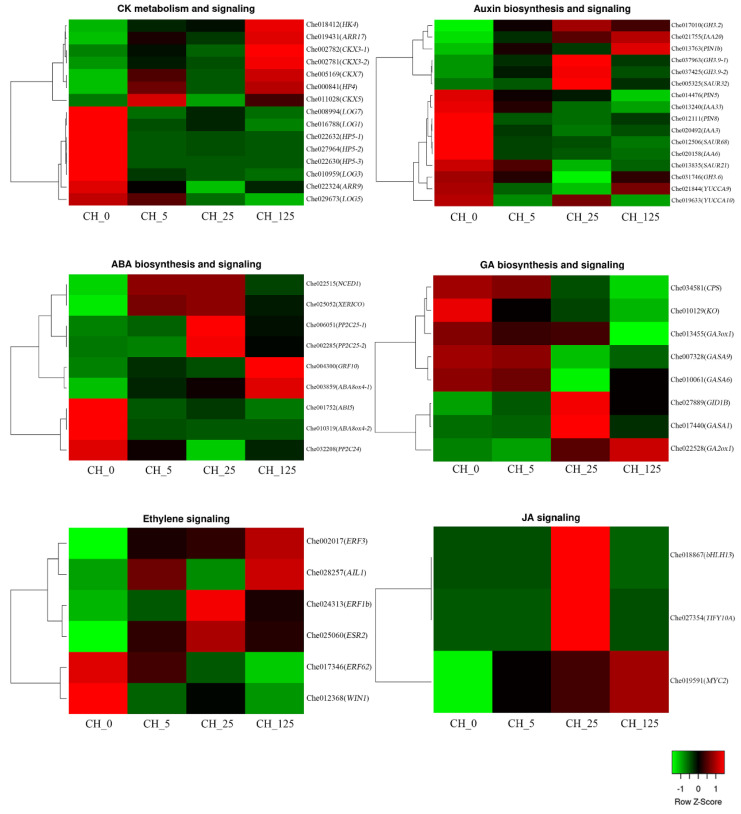
Hierarchical clustering of DEGs associated with biosynthesis, metabolism and signal transduction of cytokinin, auxin, abscisic acid, gibberellin, ethylene and jasmonate.

Many CK biosynthesis and signaling-related genes were identified as differentially expressed in response to CPPU treatment (Figure 9). *Cytokinin riboside 5′-monophosphate phosphoribohydrolases* (*LOGs*) and *cytokinin oxidases* (*CKXs*) are two key regulators of endogenous CK synthesis and metabolism. *LOGs* directly activate tZ-nucleotides and iP-nucleotides to become free CKs [13,15], whereas *CKXs* directly participate in the degradation of CKs [16,17]. In this study, four *LOG* genes, *LOG1* (Che016788), *LOG3* (Che010959), *LOG5* (Che029673) and *LOG7* (Che008994), were significantly down-regulated, and three *CKX* genes, *CKX3* (Che002782 and Che002781), *CKX5* (Che011028) and *CKX7* (Che005169), were significantly up-regulated after CPPU treatment, indicating that the content of endogenous CK has a strong feedback regulation effect after CPPU treatment.

The CK signal transduction pathway is also known as the two-component signaling system [18]. In this study, some genes involved in CK signal transduction were found to be significantly differentially expressed. The results show that *HP5* (Che022632, Che027964 and Che022630) and *ARR9* (Che022324) were significantly down-regulated, whereas *HP4* (Che000841), *HK4* (Che018412) and *ARR17* (Che019431) were significantly up-regulated after CPPU treatment.

### 2.6. DEGs Related to Endogenous Hormonal Biosynthesis and Signaling

Phytohormones such as CK, GA, ABA and auxin are key regulatory factors that control the initiation, development and sex differentiation of flowers. Moreover, the effects of CPPU treatment on biosynthesis and the signal transduction of other hormones were investigated (Figure 9). The results of the present study indicate that eight auxin biosynthesis and signaling genes were differentially expressed after CPPU treatment. In *C. henryi*, five genes, i.e., *Gretchen-Hagen3.2* (*GH3.2*, Che017010), *auxin-responsive protein IAA20* (Che021755), *auxin efflux carrier component 1b* (*PIN1b*, Che013763), *indole-3-acetic acid-amido synthetase GH3.9* (Che037963 and Che037425) and *auxin-responsive protein SAUR32* (Che005325), were up-regulated, and ten genes, *PIN5* (Che014476), *PIN8* (Che012111), *IAA3* (Che020492), *IAA6* (Che020158), *IAA33* (Che013240), *SAUR 21* (Che013835), *SAUR 68* (Che012506), *GH3.6* (Che031746), *YUCCA9* (*YUC9*, Che021844) and *YUCCA10* (*YUC10*, Che019633), were down-regulated after CPPU treatment. Furthermore, three ABA biosynthesis genes and four ABA signaling genes were changed by CPPU treatment. The expression of ABA biosynthesis-related genes *XERICO* (Che025052), *NCED1* (Che022515) and *abscisic acid 8′-hydroxylase 4* (*ABA8ox4-1*, Che003859) were significantly up-regulated, and *ABA8ox4-2* (Che010319) was significantly down-regulated by CPPU treatment. Four ABA signaling-related genes, *protein phosphatase 2C 25* (*PP2C25*, Che006051 and Che002285) and *growth-regulating factor 10* (*GRF10* and Che004300) were significantly up-regulated, and two ABA-signaling-related genes, *PP2C24* (Che032208) and *abscisic acid-insensitive 5* (*ABI5*, Che001752), were down-regulated in response to CPPU treatment. Additionally, the expression of genes involved in GA biosynthesis and signaling, including *gibberellin-regulated protein 1* (*GASA1*, Che017440), *GASA6* (Che010061), *GASA9* (Che007328), *gibberellin 2-beta-dioxygenase 1* (*GA2ox1*, Che022528), *gibberellin 3-beta-dioxygenase 1* (*GA3ox1*, Che013455), *copalyl diphosphate synthase* (*CPS*, Che034581), *ent-kaurene oxidase* (*KO*, Che010129) and *gibberellin receptor GID1B* (Che027889), were altered by CPPU treatment. The expression of several genes involved in the regulation of ETH-signaling (Che025060, Che028257, Che024313, Che012368, Che017346 and Che002017) and JA (Che019591, Che027354 and Che018867) were also altered by CPPU treatment. These results indicate that CKs may regulate floral organ development through interactions with other endogenous phytohormones in *C. henryi*.

### 2.7. Screening of Key Genes in the Hormone Biosynthesis Pathway

GAs and CKs play a leading role in the flower sex differentiation of *C. henryi* [19]. The genes related to the biosynthetic pathways of these two hormones were investigated, and the results show that the concentration of bioactive CKs is significantly positively correlated with the genes *LOG1* (Che016788), *LOG3* (Che010959) and *LOG7* (Che008994) in their metabolic pathways (Table 3), and these three genes were significantly down-regulated after CPPU treatment compared with the control group. *LOG* is a key gene family that can regulate the synthesis of CKs, and changes in its expression positively affect the concentration of bioactive CKs in *C. henryi*. The bioactive GA concentration is significantly positively correlated with *KO* (Che010129) in its biosynthetic pathway (Table 4).

### 2.8. Identification of the DEGs Related to Floral Development

**Figure 10:**
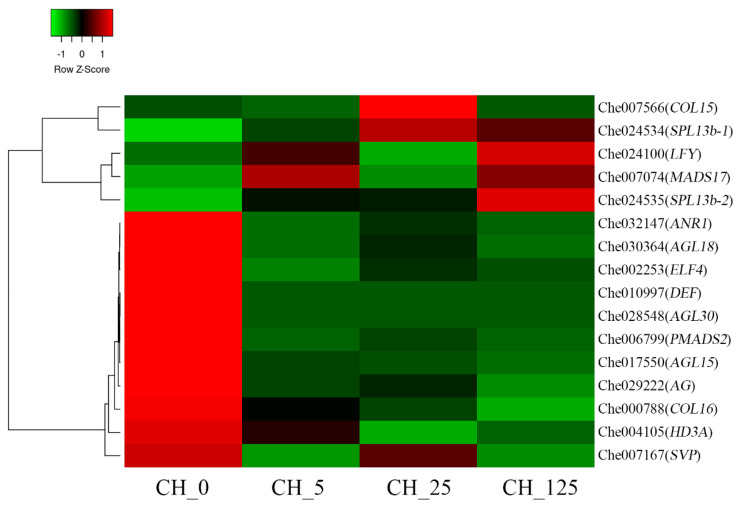
Hierarchical clustering of DEGs involved in the regulation of floral organ and floral development after N-(2-chloro-4-pyridyl)-N’-phenylurea (CPPU) treatment in *Castanea henryi*.

The development of floral organs of typical higher plants can be divided into four rounds, with different genes regulating the floral organs in each round. Most ABC floral organ regulator genes belong to the MADS-box gene family [20,21]. Transcriptome data show that 16 genes related to flower development responded to CPPU treatment (Figure 10). Four genes belonged to the MADS-box gene family: *MADS-box transcription factor 17* (*MADS17*, Che007074), *floral homeotic protein PMADS 2* (Che006799), *MADS-box transcription factor ANR1* (Che032147) and *MADS-box protein SVP* (Che007167). B-class Mads-box gene *floral homeotic protein DEFICIENS* (*DEF*, Che010997), C-class Mads-box gene *floral homeotic protein AGAMOUS* (*AG*, Che029222), D-class Mads-box genes *agamous-like 15* (*AGL15*, Che017550), *AGL18* (Che030364) and *AGL30* (Che028548) were differentially expressed after CPPU treatment. Furthermore, *early flowering 4* (*ELF4*, Che002253), a gene related to early floral meristem development, was differentially regulated by the CPPU treatment. Moreover, four genes that control the timing of flower formation, namely *squamosa promoter-binding-like protein 13b* (*SPL13b*, Che024534 and Che024535), *zinc finger protein constans-like 15 (COL15*, Che007566), *COL16* (Che000788), *leafy-like protein* (*LFY*, Che024100) and *protein HEADING DATE 3A* (*HD3A*, Che004105), were also differentially expressed after CPPU treatment. In conclusion, the identification of floral organ properties provides a theoretical basis for understanding sex determination in *C. henryi.*

## 3. Discussion

### 3.1. CPPU Has Strong Feminization Effects on the Floral Development in C. henryi

To increase the yield of monoecious plants, it is important to increase the ratio of male to female flowers. Phytohormones such as CK [22], GA [23], auxin [24] and ETH [25] promote the conversion of male flowers into female flowers in different plants. Exogenous CK spraying has been reported to alter the ratio of male to female flowers in *Actinidia chinensis* [26], *Jatropha curcas* [6] and *Mercurialis annua* [7]. The results of the present study on *C. henryi* are consistent with these findings, as CK treatment showed strong feminization effects on floral development. This suggests that an astonishing 100% conversion rate can be achieved with appropriate treatment.

These results suggest that CK is a key regulator controlling female flower development as well as a potential determinant of sex differentiation in *C. henryi*. In addition, *C. henryi* was observed to be less sensitive to low concentrations of CPPU, with the conversion of only some of the male flowers into female flowers, which was significantly lower than the promotional effect of high concentrations. Therefore, it is suggested that low concentrations of CPPU are insufficient for the complete conversion of male flowers to female flowers. However, exogenous CPPU treatment was still effective in inducing the conversion of male flowers to female flowers. Transcriptome data analysis of different treatment groups can provide precious information for studying the gene network of CKs that regulate sex differentiation in *C. henryi*.

### 3.2. Exogenous Cytokinin Treatment Alters Endogenous Cytokinin Levels and the Expression of Cytokinin Biosynthesis and Signaling-Related Genes

In plants, CKs exist in various forms, and the supply of exogenous CKs affects endogenous cytokinin content [27]. In this study, exogenous cytokinin (CPPU) treatment was observed to significantly reduce the level of endogenous cytokinin, which may be due to the presence of bioactive cytokinin in other bound forms, reducing endogenous cytokinin levels. Furthermore, genes related to the CK metabolic pathway were identified, and the results show that the expression levels of three genes (*CKX3*, *CKX7* and *CKX5*) of the *CKX* gene family, which are involved in CK degradation and the regulation of CK homeostasis in plants [28,29], are negatively correlated with cytokinin levels. The expression levels of three genes (*LOG7*, *LOG1* and *LOG3)* of the *LOG* gene family, which encode a 5′-ribose monophosphate hydrolase that directly converts iPRMP and tZRMP to iP and tZ [13], are significantly positively correlated with CK levels. The results show that the exogenous CPPU treatment of *C. henryi* altered the expression of genes related to the CK metabolic pathway, which in turn affected the levels of endogenous CK. In *LOG* mutants, rice panicles were severely reduced in size, and branching patterns were abnormal. This is accompanied by the abnormal development of floral organs, with flowers having only one stamen and no pistil [15]. Hence, it was inferred that the decrease in the relative expression of *LOG* in the treatment group may affect the normal formation of stamens and promote female flower differentiation.

**Figure 11:**
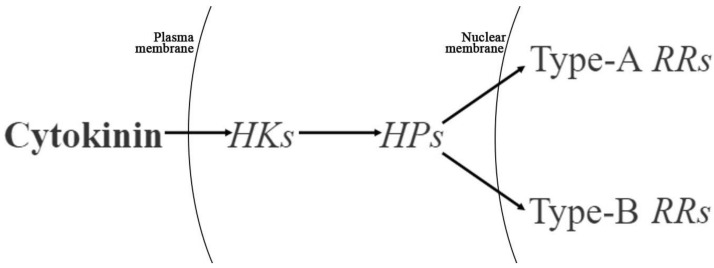
Model of cytokinin signaling (Adapted from To et al. [32]).

In the signaling model of CK, it binds to the receptor of *histidine kinases* (*HKs*) and transfers the phosphate group to downstream *histidine transfer proteins* (*HPs*), which in turn transfers the phosphate group to type-A *response regulators* (type-A *RRs*) or type-B *response regulators* (type-B *RRs*) [30] (Figure 11). Type-A and type-B *RRs* regulate the transcription of CK-responsive genes, thereby regulating plant growth and development [30,31]. In this study, five genes were differentially expressed in the signal transduction pathway. Among them, the relative expression of *HK4*, *ARR17* and *HP4* in the treatment group was significantly higher than it was in the control group, indicating that the activities of genes related to the CK signaling pathway in female flowers were higher than those in male flowers.

### 3.3. Exogenous CK Treatment Alters Endogenous GA Levels and the Expression of GA Biosynthesis and Signaling-Related Genes

GA has been demonstrated to be a key hormone that promotes male flower formation in *C. henryi* [19]. In this study, the GA content in the treatment group was significantly lower than that in the control group. Moreover, the GA content showed a decreasing trend with increasing CPPU concentrations, which was consistent with the increase in the proportion of female flowers. The significantly decreased expression levels of *CPS*, *KO* and *GA3ox* and the increased expression levels of *GA2ox* may be the reason for the decreased endogenous GA levels. *Ent-kaurene oxidase KO* is a single-gene regulatory enzyme that functions upstream of GA synthesis. It is a cytochrome P450 and NADPH-dependent monooxygenase belonging to the CYP701 subfamily that catalyzes the final formation of ent-kaurene acid by catalyzing ent-kaurene C19 [33]. The down-regulation of *AtKO1* in transgenic *Arabidopsis* results in a decrease in GA_4_ content [34]. Tissue-specific studies have shown that *KO* gene expression exhibits distinct spatial differences [35]. The gene *AtKO* in *Arabidopsis* is expressed in all tissues, with the highest expression observed in the inflorescence. In this study, the gene *KO* showed the highest relative expression in male catkins of the control group and is significantly positively correlated with GA levels, implying that it affects the formation of catkins.

The gene *GASA* is one of the few identified target genes downstream of the GA signaling pathway [36,37], and it is mostly expressed strongly in young plant tissues, organs and parts of vigorous growth [38,39]. In *Arabidopsis*, *GASA* participates in the regulation of floral meristem decisions, promotes flowering [40] and regulates GA by promoting GA signaling and inhibiting redox activity to promote seed germination [36]. In this study, three members of the *GASA* gene family were identified as DEGs, indicating that they affect plant hormone signal transduction.

### 3.4. Exogenous CK Treatment Alters the Levels of Other Endogenous Phytohormones

CK coordinates plant growth and development with phytohormones, such as auxin, ABA, ETH and JA, in a synergistic or antagonistic manner [41]. Previous studies have shown that CKs and auxins regulate their biosynthesis and signal transduction during plant growth and development [42]. In this study, although 15 genes related to auxin biosynthesis and signal transduction pathways were differentially expressed, there was no significant difference in auxin content in the treatment groups, possibly owing to the time delay between gene expression and protein synthesis [43]. The role of ABA in sex differentiation differs among plants. In this study, ABA levels showed no clear trend among the treatment groups, suggesting that endogenous ABA levels were not significantly affected by the exogenous CK treatment. CK also interacts with ETH and JA to regulate flowering, and genes related to the two hormones’ signal transduction pathways were found to be differentially expressed in this study. It is worth mentioning that the gene *MYC2*, identified in the JA signaling pathway, is a “master switch” in the crosstalk between JA and the other hormone signaling pathways, and it was significantly up-regulated after CPPU treatment. These results indicate that CKs interact with auxin, ABA, GA, ETH and JA to determine the sex of *C. henryi*. Moreover, these findings suggest that CK determines endogenous hormone levels in *C. henryi* by altering the expression levels of genes involved in the synthesis and transduction of endogenous CK, GA, auxin, ABA, ethylene and JA, ultimately altering the sex of *C. henryi* flowers.

### 3.5. CK Regulated the Expression of Genes Related to Floral Organ Development

Studies have shown that *DEFICIENS* (*DEF*), *PISTILLATA* (*PI*) and *AGAMOUS* (*AG*) jointly control stamen formation [44]. Mandel et al. found that the absence of the *AP3* (homologous to *DEF*) gene product in *Arabidopsis* causes petals to be replaced by a ring-shaped sepal, and it causes stamens to become carpels [45]. Mutations or the ectopic expression of the *AG* gene has demonstrated its decisive role in stamen and carpel development [45]. In this study, the expression of *DEF* and *AG* genes in each treatment group was significantly down-regulated, which may have affected the normal formation of stamens, resulting in a significant decrease in the number of stamens compared to the control group. *LFY* plays an important role in activating gene activity in floral organs. In the *LFY* mutant of *Arabidopsis*, the floral organ-determining gene *AP3* was decreased in both the expression region and expression level [46]. *LFY* and *WUS* act together to activate the expression of *AG* and simultaneously act with *UNUSUAL FLORAL ORGANS* (*UFO*) to activate the expression of *AP3*, thereby forming floral organs [47]. In this study, the *LFY* gene was significantly up-regulated in the treatment groups, presumably indicating its critical role in processes involved in flower sex expression. In rice *OsMADS1* gene transformants, the number of stamens was reduced [48]. In this study, the *MADS17* gene, which is a direct downstream gene of *MADS1* [49], was significantly differentially expressed in the treatment group. Therefore, it is speculated that the differential expression of the *MADS17* gene may be the reason for the decreased number of stamens in the treatment group.

The gene *PMADS2* was only expressed in the petals and stamens of *Petunia* [50]. In this study, the expression of *PMADS2* was significantly down-regulated, and the number of male flowers in the treatment group was lower than it was in the control group, indicating that the decrease in its expression may affect the development of flowers. The gene *SVP* can inhibit normal flowering in plants [51]. With an increase in the number of female flowers in the treatment group, the relative expression of the *SVP* gene decreased, which gradually reduced the inhibitory effect on *C. henryi* flowering and may be beneficial to the development of pistils. The gene *AGL15* is involved in various in vivo regulatory pathways. The gene downstream of *AGL15* in *Arabidopsis* is a key enzyme in the GA regulatory pathway [52]. In the present study, the expression of *AGL15* was significantly down-regulated in the treatment group. It is speculated that this affected the related regulatory pathway of GA, which in turn changed the number of pistils. This is consistent with the results of the present study. These results suggest that CKs regulate sex differentiation in *C. henryi* by directly targeting genes associated with androecium and gynoecium development.

### 3.6. Regulatory Networks Participate in Promoting Floral Feminization in C. henryi

**Figure 12:**
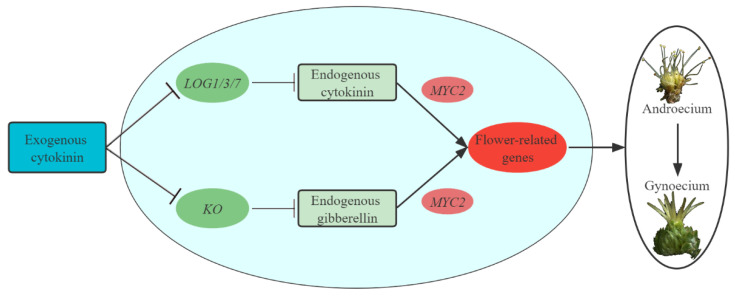
A schematic model for the role of cytokinin in the regulation of sex determination in *Castanea henryi*. Arrows and blunt arrows represent positive and negative regulations, respectively.

In the present study, many genes involved in CK, GA, auxin, ABA, ETH and JA biosynthesis and signal transduction were identified, suggesting that these hormones may be involved in the process of feminization of the male flowers of *C. henryi*. Particularly, we speculate that the endogenous cytokinin and gibberellin were the key phytohormones that promoted the transformation of male to female flowers in *C. henryi*, and the expression of genes *LOG1*/*3*/*7* and *KO* were the key factors that altered the levels of both phytohormones. Therefore, it would be interesting to investigate how the interaction of these hormones feminizes the male flower of *C. henryi*. *MYC2*, which was identified in the JA signaling pathway, has a broad role in regulating developmental programs and controls crosstalk between JA and nearly all the other hormone signaling pathways [53]. *MYC2* is not only implicated in cytokinin signaling by interacting with *HP5* [54] and *HP6* [55] in the cytokinin signaling pathway, but it also transduces the GA signaling to the biosynthesis of the sesquiterpenes pathway by interacting with *DELLA* [56]. Based on the results, we speculate that *MYC2* crosstalks the transduction of CK and GA signaling, which may lead to mutations in one or more genes associated with floral organ development, ultimately causing the transition of androecium to gynoecium in *C. henryi* (Figure 12). Whether the genes mentioned in the regulatory network are the key target genes to improve the yield of *C. henryi* remains to be our further study.

## 4. Materials and Methods

### 4.1. Plant Materials, Growth Conditions and Treatment

The *C. henryi* “Huali 4” cultivar was obtained from the Central South University of Forestry and Technology (28°818′′N, 113°015′′E), Hunan Province, China. Catkins were selected from three 11-year-old trees that were planted with 3 m × 2.5 m spacing, pruned and fertilized every year in December.

A certain amount of CPPU (5 mg CPPU for 5 mg·L^−1^, 25 mg CPPU for 25 mg·L^−1^ and 125 mg CPPU for 125 mg·L^−1^) was dissolved in 25 mL absolute ethanol to prepare a plant hormone stock solution. Stock solutions were diluted with 1 L of pure water to prepare working solutions of different concentrations. As shown in Figure 1a, the catkins were treated with working solutions one week after the buds sprouted for the first time, when the length of the bud was approximately 1.5 cm (March 22). Three concentrations of CPPU, A (5 mg·L^−1^), B (25 mg·L^−1^) and C (125 mg·L^−1^), were applied at three different periods (the interval is one week in 2021) with a 500 mL plastic sprayer to the *C. henryi* catkins. At each concentration, CPPU was applied to the *C. henryi* catkins in seven different ways, and each treatment is represented as A1, B1, C1, …, C7. Three biological replicates were used for each treatment, and the control group CK was treated with pure water instead of hormones.

### 4.2. Sample Collection

Two weeks after the third spraying, when the carpel and stamen primordia began to differentiate [19,57] (April 20), the male catkins of treatment groups CK, A7, B7 and C7 (see Table 5 for details) were collected as samples and were named CH_0, CH_5, CH_25, and CH_125, respectively. They were then snap-frozen in liquid nitrogen and were stored in a −80 °C freezer for endogenous hormone concentration determination, transcriptome sequencing and quantitative real-time polymerase chain reaction (RT-qPCR).

Total RNA was extracted using the Plant Total RNA Isolation Kit (Omega, China), and the concentrations and purities of RNA were detected using a UV spectrophotometer and NanoDrop 2000c spectrophotometer (Thermo Fisher, Waltham, MA, USA). A sequencing library construction for each sample with three biological replicates was completed by Igenebook Biotechnology Co., Ltd. (Wuhan, China).

### 4.3. Determination of Endogenous Hormones

The levels of endogenous hormones iP, iPR, tZ, tZR, GA_1_, GA_4_, IAA, ABA and JA in the male catkin of *C. henryi* in each treatment were determined using high-performance liquid chromatography–mass spectrometry (HPLC-MS) [58]. An Agilent 1290 HPLC system (Agilent) and a Qtrap 6500 mass spectrometer (Sciex) were used for quantification. Standards were purchased from Sigma-Aldrich. A reversed-phase poroshell 120 SB-C18 column (2.1 × 150 mm, 2.7 μm) was used as the stationary phase, and the mobile phase was a solution of A: B = (methanol/0.1% formic acid): (water/0.1% formic acid). The column temperature was 30 °C, and the sample volume was 2 μL. The ionization mode was negative. Other parameters detected by mass spectrometry were: curtain gas pressure, 15 psi; spray voltage, −4000 V; nebulizing gas pressure, 65 psi; auxiliary gas pressure, 70 psi; and nebulizing temperature, 400 °C.

### 4.4. Illumina Transcriptome Sequencing and Assembly of Clean Reads

Base calling was used to convert the raw image data files obtained by high-throughput sequencing into raw sequenced reads, namely raw reads or raw data. The original data were filtered and processed to obtain clean reads as follows [59]: the clean reads data were aligned to the reference genome using HISAT2 software [60] (version: 2.0.1-beta); the transcripts of all samples were reconstructed from the alignment information to obtain total reads using StringTie software [61] (version: 2.0.4); and the number of reads of the gene was normalized by the fragments per kilobase of exon per million reads mapped (FPKM) normalization method [62,63]. The genome was divided into five regions: CDS, 5UTR, 3UTR, intron and intergenic, and the total reads aligned to the genome were counted according to the functional elements. Most RNA-seq reads fall within the CDS region under normal conditions. Based on the expression level of each sample gene, the correlation between samples was calculated using the Pearson correlation coefficient. Genes with FDR < 0.05 and |FoldChange| > 2 were considered significantly different genes. Differential expression analysis was performed using edgeR [64]. The expression levels of significantly different genes in all samples were extracted into a list, and the Pheatmap function was used to draw a heat map of the DEGs.

### 4.5. Transcript Annotation and Gene Expression Analysis

The functional annotation of DEGs was performed against multiple nucleic acids and protein public databases: GO annotation (Gene Ontology) [65,66]; Nr annotation (NCBI Non-Redundant Protein Sequences); and KO annotation (KEGG Ortho Database) [67,68,69]. To quantify the DEGs, the FPKM method was used to calculate read counts as the number of fragments per kilobase of the transcript [70].

### 4.6. Validation of DEGs by RT-qPCR

To confirm the Illumina sequencing results, 10 candidate genes were randomly selected, and the expression of the DEGs in three different samples was verified using RT-qPCR. cDNA for RT-qPCR was synthesized according to the instructions of HiScript II Q RT SuperMix (Vazyme Biotechnology, Nanjing, China). The total volume of the qPCR reaction system was 20 μL, including 10 μL of 2 × ChamQ Universal SYBR qPCR Master Mix (Vazyme Biotechnology, Nanjing, China), each with 0.4 μmol/L of forward and reverse primers, 1 μL of ten-fold diluted cDNA template and 8.2 μL ddH_2_O. The PCR conditions were as follows: denaturation at 95 °C for 30 s; 40 cycles of denaturation at 95 °C for 10 s; and annealing and extension at 60 °C for 30 s. Gene expression was calculated using the 2^−ΔΔCt^ method [71]. The primer sequences of the reference gene (GAPDH) and 9 selected genes are shown in Table 2.

### 4.7. Statistical Analysis

Data were analyzed using Statistical Product and Service Solution (SPSS) software (version 13.0; SPSS, Chicago, IL, USA). The means were compared using Student’s *t*-test at the 5% significance level. Three replicates were performed for each treatment. Figures were generated using OriginPro (Microcal Software Inc., Northampton, MA, USA).

## 5. Conclusions

In conclusion, this study demonstrates that the exogenous administration of CPPU has a strong feminization effect on *C. henryi*. Transcriptome analysis of the treatment group, in which male flowers were converted to female flowers after CPPU treatment, helped to identify candidate genes that determine sex regulation. As was expected, these candidate genes have only been shown to be related to the sex of flowers in some plants, and whether they have the same effect in *C. henryi* requires further study. This study provides a basis for further understanding the molecular mechanisms of CK regulation in sex determination in *C. henryi*.

## Figures and Tables

**Table 1:** The statistics of reads before and after filtering.

Sample	RawReads	RawBases	CleanReads	CleanBases	CleanRatio	Q20	Q30	GC
CH_0_1	66,611,830	9.99 × 10^9^	66,065,838	9.67 × 10^9^	99.18%	97.81%	93.75%	45.35%
CH_0_2	66,288,344	9.94 × 10^9^	65,736,958	9.63 × 10^9^	99.17%	97.65%	93.33%	45.51%
CH_0_3	60,326,280	9.05 × 10^9^	59,880,596	8.67 × 10^9^	99.26%	97.84%	93.78%	45.05%
CH_5_1	57,234,618	8.59 × 10^9^	56,636,914	8.32 × 10^9^	98.96%	97.41%	92.83%	46.13%
CH_5_2	56,737,570	8.51 × 10^9^	56,058,944	8.31 × 10^9^	98.80%	97.23%	92.51%	45.81%
CH_5_3	60,847,540	9.13 × 10^9^	60,135,430	8.83 × 10^9^	98.83%	97.35%	92.75%	45.96%
CH_25_1	65,534,342	9.83 × 10^9^	64,933,306	9.49 × 10^9^	99.08%	97.63%	93.35%	45.33%
CH_25_2	64,267,422	9.64 × 10^9^	63,506,822	9.29 × 10^9^	98.82%	97.50%	93.10%	45.98%
CH_25_3	48,063,476	7.21 × 10^9^	47,646,260	6.97 × 10^9^	99.13%	97.76%	93.52%	43.58%
CH_125_1	61,642,840	9.25 × 10^9^	60,986,388	8.94 × 10^9^	98.94%	97.57%	93.21%	46.12%
CH_125_2	61,918,812	9.29 × 10^9^	61,382,232	9.03 × 10^9^	99.13%	97.68%	93.43%	45.97%
CH_125_3	64,129,072	9.62 × 10^9^	63,554,386	9.37 × 10^9^	99.10%	97.65%	93.41%	45.73%

**Table 2:** Details of oligonucleotide primers used for quantitative real-time polymerase chain reaction (RT-qPCR).

Gene ID	Direction	Primer sequence (5′–3′)	Annealing Temperature (°C)	Amplicon (bp)
Che006645	Forward	GGAGTTCAAGAACCGGACACCATC	54.8	85
	Reverse	CAAGCAAACCGAGCATTCATGTTCC		
Che017010	Forward	CGAAGACGATTCCTGGTCACTACG	54.7	81
	Reverse	GTACTTCCTCACTTGGCGAGTTAGC		
Che020159	Forward	CGGTGAGAGCATCAAGGAAGAACG	55.9	81
	Reverse	CCCCATTTGCAGGGTCCATAAGC		
Che019063	Forward	AACAACGCCTCCAAGCTCTAATCG	54.9	142
	Reverse	GCCTTATCGTCCTCGCCTTTGTAG		
Che001701	Forward	CAGTGGCAGGAGCTTGAACTACAG	55.2	131
	Reverse	AGAGGGTGATGGAGGAAGTAAGGTG		
Che002781	Forward	ATGGCTCGTAATGGGGTTGTTGTG	55.9	87
	Reverse	TGACGGGGTTCCAGAGACAGTG		
Che004615	Forward	CGTCATTGGGGTTCATGGGTATCC	55.4	89
	Reverse	GCCTCTTCAGCAGTCTCGAATGTG		
Che008994	Forward	TTTATCTCGCCAACCGCACGTC	55.8	126
	Reverse	CACTCCTTCCCAAACCAGCTTCG		
Che016788	Forward	GAGGTTGCCATGTTCTCGGAGTG	55.1	118
	Reverse	GCCATTTCTGCCTTCCTTTGATGC		
GAPDH	Forward	AGCAAGGACTGGAGAGGTGGAAG	56.1	136
	Reverse	CGGTAGGAACACGGAAAGCCATC		

**Table 3:** Correlations between the concentrations of cytokinin and its metabolism-related genes.

Variable	*CKX3-1*	*CKX3-2*	*CKX7*	*CKX5*	*LOG1*	*LOG3*	*LOG7*	*LOG5*
tZ + iP	−0.58	−0.66	−0.80	−0.47	0.991 **	0.996 **	0.987 *	0.79

Notes: * *p* < 0.05, ** *p* < 0.01. *CKX3-1* (Che002782), *CKX3-2* (Che002781), *CKX7* (Che005169), *CKX5* (Che011028), *LOG1* (Che016788), *LOG3* (Che010959), *LOG7* (Che008994) and *LOG5* (Che029673). The numbers represent the Pearson correlation coefficients, with negative values being negative correlations and positive values being positive ones.

**Table 4:** Correlations between the concentrations of gibberellin and its metabolism-related genes.

Variable	*KO*	*CPS*	*GA2ox1*	*GA3ox1*
GA_1_ + GA_4_	0.973 *	0.81	−0.74	0.62

Notes: * *p* < 0.05, *KO* (Che010129), *CPS* (Che034581), *GA2ox1* (Che022528) and *GA3ox1* (Che013455). The numbers represent the Pearson correlation coefficients, with negative values being negative correlations and positive values being positive ones.

**Table 5:** Different treatment methods.

A (5 mg·L^−1^)				B (25 mg·L^−1^)				C (125 mg·L^−1^)			
	3.22	3.29	4.5		3.22	3.29	4.5		3.22	3.29	4.5
1	√			1	√			1	√		
2		√		2		√		2		√	
3			√	3			√	3			√
4	√	√		4	√	√		4	√	√	
5		√	√	5		√	√	5		√	√
6	√		√	6	√		√	6	√		√
7	√	√	√	7	√	√	√	7	√	√	√

Notes: The capital letters A, B, and C indicate different N-(2-chloro-4-pyridyl)-N’-phenylurea (CPPU) treatment concentrations, the numbers 1–7 indicate different treatment times of CPPU, the marked place is the treatment time under the CPPU concentration and different treatment groups are named A1, B1, C1, …, A7, B7, C7.

## Data Availability

The data of the RNA-seq of *Castanea henryi* have been deposited in NCBI (BioProject: PRJNA818183).
